# Highly sensitive and selective detection of naproxen *via* molecularly imprinted carbon dots as a fluorescent sensor†

**DOI:** 10.1039/d1ra04817a

**Published:** 2021-08-31

**Authors:** Ke Li, Min Zhang, Xingyu Ye, Yongming Zhang, Guisheng Li, Rui Fu, Xiaofeng Chen

**Affiliations:** School of Environmental and Geographical Sciences, Shanghai Normal University Shanghai 200234 China xiaofengchen@shnu.edu.cn; Education Ministry Key and International Joint Lab of Resource Chemistry, Shanghai Key Laboratory of Rare Earth Functional Materials, Shanghai Normal University Shanghai 200234 China; RNA Bioscience Initiative, University of Colorado School of Medicine Aurora CO 80045 USA

## Abstract

The overuse and inappropriate discharge of naproxen, a common anti-inflammatory medication and an emerging contaminant in water, is detrimental to human health and bodies of water. Here, we design a fluorescent sensor based on molecularly imprinted carbon dots (CDs) for highly selective detection of trace amounts of naproxen. The CDs were encapsulated into the pores of silica through a sol–gel based method and provide fluorescent signal. After removal of the template molecules, a molecularly imprinted polymer layer was formed and the fluorescence of the CDs sensor was selectively quenched by naproxen. A detection limit of as low as 0.03 μM and a linear range of 0.05–4 μM for detecting naproxen in aqueous solution were obtained. High recoveries of naproxen levels in waste water and urine samples for practical application were also achieved. In addition, the accurate detection performance of sensor was maintained during the UV degradation of naproxen.

## Abstract 2

A highly selective fluorescent sensor for naproxen utilizes carbon dots as the fluorophore and molecularly imprinted polymer to provide the recognition sites. The fluorescence of carbon dots can be selectively quenched by naproxen.

## Graphical abstract

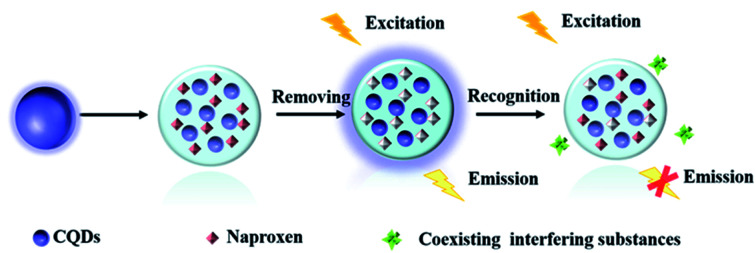

## 1. Introduction

Naproxen, one of the four most commonly used nonprescription drugs, is often used to relieve inflammation, fever, and pain.^1^ However, prolonged and excessive use of naproxen leads to side effects such as gastrointestinal bleeding, stomach ulcers, and increased risk of heart disease.^2^ In addition, naproxen can be released into domestic sewage processing system *via* bodily excretions, and linger within natural water bodies due to its high chemical stability. The existence of naproxen impacts bivalve lipid peroxidation, microbial community structure, and cause antibiotic resistance.^3^ The high polarity and low volatility of naproxen further promote expansion of pollution in water. Therefore, developing rapid, simple, and effective detection methods of naproxen is highly desirable. Traditional approaches for analysing the existence of naproxen include electrochemical techniques,^4,5^ high performance liquid chromatography(HPLC),^6^ flow injection analysis,^7^ magnetic-solid phase extraction (M-SPE),^8^ capillary electrophoresis (CE),^9^ and absorption spectrophotometry.^10^ These methods, however, demand specialized training, costly instruments, and complex sample preprocessing. Fast detection of naproxen in water is of great significance. As an intriguing alternative to analytical methods, fluorescence sensing has attracted increasing attentions in recent years, due to its high sensitivity and fast response. Fluorescent sensors have been widely developed in the detection of pollutants such as Hg^2+^,^11^ Pb^2+^,^12^ Cd^2+^,^13^ Ag^+^,^14^ perfluorooctane sulfonate,^15^ bisphenol A,^16^ and phosphate.^17^

Compared to organic fluorophores, inorganic quantum dots possess desirable optical properties of broad excitation spectrum, narrow emission peak, and light bleaching resistance.^18^ Traditional quantum dots, such as CdS^19^ and CdTe,^20^ are commonly based on rare metals. Recently, carbon dots have been widely exploited for applications since they are easy to prepare, cost-effective and environmentally friendly. Fluorescent carbon dots in particular benefit from their light stability, photoluminescence, and size-dependent emission wavelength,^21^ and have been extensively utilized in the field of photocatalysis,^22^ cell imaging,^23^ biosensors,^24–26^ and more. Carbon dots have been developed rapidly for fluorescent sensors,^27,28^ however, they still largely serve as fluorescence carrier with limited selectivity.^29,30^

To improve the selectivity of carbon dots-based sensors, molecular imprinting technique has been introduced to provide recognition sites for the analyte. Molecularly imprinted polymers (MIP) are the functionalized polymers formed in the copolymerization process of functional monomers, crosslinking agents and target template molecules.^31^ After removal of the template molecules, imprinted cavities of the exactly same shape and size complementary to target molecules are obtained, achieving specific recognition ability towards target molecules.^32–35^ Given the high specificity, molecularly imprinted polymers have found great potentials in sensor applications.^36–38^ Recent advances in combining fluorescent quantum dots with molecular imprinting polymer have dramatically improved the selectivity of quantum dot sensors,^39,40^ in the case of detecting metronidazole,^41^ amoxicillin,^42^ promethazine hydrochloride,^43^ cocaine,^44^ and doxorubicin.^45^ However, to the best of our knowledge, detection of naproxen based on molecularly imprinted carbon dots has not been reported to date.

**Scheme 1:**
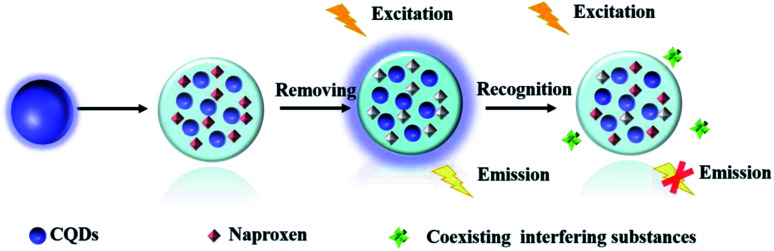
Illustration of the preparation and fluorescence response of MIP@CDs to naproxen.

Herein, a highly selective fluorescent sensor (MIP@CDs) for naproxen was developed, utilizing carbon dots as the fluorophore and molecularly imprinted polymer to provide the recognition sites. As shown in Scheme 1, carbon dots were embedded in silica imprinted film through one-step sol–gel polymerization. After the removal of template molecules, the recognition sites were then released. The fluorescence of carbon dots was selectively quenched by naproxen, with a low detection limit of 0.03 μM and a linear range of 0.05–4 μM.

## 2. Experimental

### 2.1 Chemicals and reagents

All chemicals are of analytical reagent grade and were used as-received without further purification. Citric acid, ammonia water, methanol, ethyl alcohol 3-(2-aminoethylamino)-propyldimethoxymethylsilane(AEAP),(3aminopropyl)triethoxysilane (APTES), tetraethyl orthosilicate (TEOS), naproxen, ibuprofen, ketoprofen, Fenoprofen were obtained from Saen Chemical Technology (Shanghai) Co., Ltd. Millipore Milli-Q ultrapure water was used for all the experiments in the work.

### 2.2 Characterization and measurements

UV-vis absorption spectra were collected with a ultraviolet-visible spectrophotometer (UV-2450, Shimadzu). Photoluminescence spectra were recorded using a fluorescence spectrometer (Fluoro-Max-4, Horiba Scientific). Scanning electron microscopy (SEM) images were obtained using an emission scanning electron microscope (S 4800, Hitachi). Fourier transform infrared (FT-IR) measurement was carried out with a FT-IR spectrometer (Nicolet iS10, Thermo Scientific). High-performance liquid chromatography (HPLC) was carried out using a UltiMate 3000 pump (Thermo Scientific). All separations were conducted on a Thermo Scientific ZORBAX SB-C18 (5 m, 4.6 mm × 150 mm, Agilent) column. Methanol and 0.2% formic acid solution (80/20 volume ratio) comprised the mobile phase. The detection wavelength was 242 nm, the flow rate was 0.2 mL min^−1^, and the column temperature was set at 40 °C. The sample was filtered through a membrane filter with a diameter of 0.22 μm before testing. All optical measurements were carried out at ambient temperature.

### 2.3 Synthesis of CDs

Carbon dots were prepared by hydrothermal method according to the reported literature with slight modifications.^46^ Briefly, 0.5 g citric acid anhydrous was dissolved in 10 mL deionized water using ultrasonator and degased in the atmosphere of nitrogen for 15 min. 2 mL AEAP was then added under vigorous stirring for 10 min. The solution mixture was transferred to a 50 mL Teflon-lined stainless steel autoclave and was kept at 200 °C for 2 h. After the reaction was completed, the autoclave was cooled to room temperature. The resultant dark brown transparent liquid was filtered through filtration membrane filter (0.22 μm) and washed with petroleum ether three times. The obtained CDs were stored in the refrigerator at 4 °C for further use.

### 2.4 Synthesis of the MIP@CDs

100 mg naproxen was dissolved in 30 mL ethanol by using ultrasonator, 0.4 mL APTES and 0.5 mL CDs were added to the solution respectively under stirring for 10 min. 1 mL TEOS was added dropwise, followed by the addition of 1 mL ammonia water. The solution mixture was then kept stirring for 24 h in the dark. The resultant product was then collected by centrifugation and washed thoroughly with methanol until no naproxen template molecules were detected from the washing solvent using ultraviolet-visible spectrophotometer. The obtained molecularly imprinted carbon dots (MIP@CDs) were then dried in vacuum oven for 24 h to get white powder product and stored in the refrigerator at 4 °C. The non-imprinted CDs (NIP@CDs) were also prepared identically without adding any naproxen.

### 2.5 Detection of naproxen

The obtained white powder of MIP@CDs was dispersed into ultrapure water to make the stock solution (280 mg L^−1^). 500 μL of stock solution and 200 μL of phosphate buffer saline (pH = 5.5, 0.1 M) were added to the cuvette, the solution volume was adjusted to 3 mL using ultrapure water. Then various amount of naproxen solution (50 μM) was introduced to the cuvette gradually. After incubation for 10 min, the fluorescence spectra was collected under the excitation wavelength of 360 nm.

### 2.6 Detection of naproxen in real water samples

Tap water, surface river water, and prepared human urine samples were selected to investigate the practical application for naproxen detection of MIP@CDs. Tap water sample was directly taken form the tap in the lab, and the surface river water sample was collected from local Caohejing River (Shanghai, China). Both of the water samples were filtered through microporous membrane (0.22 μm) to remove particulate matter before further use and monitored by HPLC to confirm the inexistence of naproxen. The urine sample was prepared according to the recipe in Table S1.† Different concentrations of naproxen were prepared using those water samples to study the fluorescent sensing behaviour of MIP@CDs. For UV degradation experiments, 30 mL of naproxen solution (10 mg L^−1^, 20 mg L^−1^, and 10 mg L^−1^, respectively) was placed in Petri dishes under UV light, and samples were taken every 15 min for a total of 3 h.

## 3. Results and discussion

### 3.1 Characterization of the structure and spectra properties of MIP@CDs

**Fig. 1:**
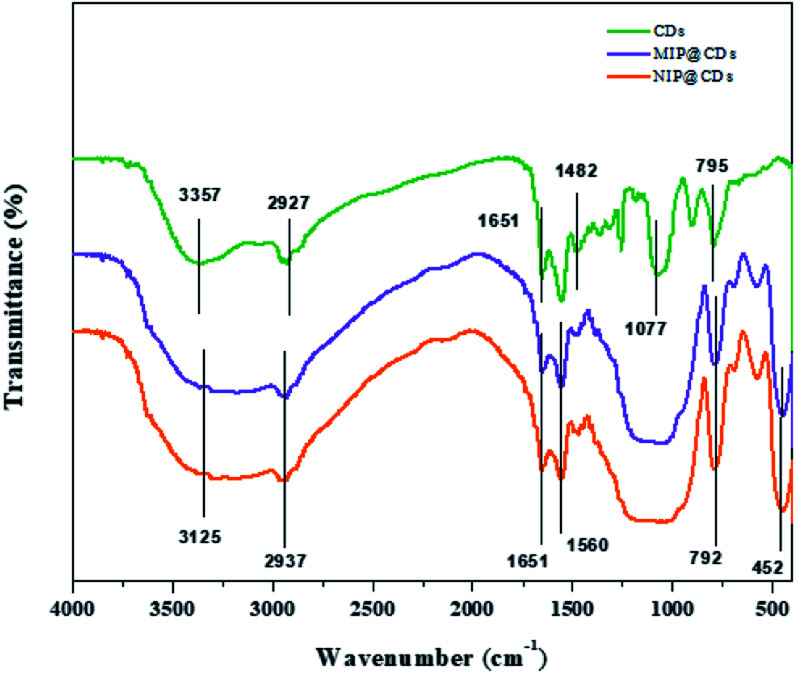
FT-IR spectra of CDs, MIP@CDs, and NIP@CDs.

The morphological structures of MIP@CDs and NIP@CDs were characterized by SEM and TEM techniques. Both MIP@CDs and NIP@CDs exhibit aggregated spherical structure with average diameters of about 50 nm (Fig. S1†). In order to further confirm the formation of imprinted polymer layer, FTIR spectra of CDs, MIP@CDs and NIP@CDs were collected. As shown in Fig. 1, the characteristic peaks at 3357 cm^−1^ and 795 cm^−1^ for CDs can be assigned to the O–H stretching and bending vibration, accounting for the formation of hydrogen bond on the surface of CDs with AEAP. The vibration signals of C=O at 1482 cm^−1^ and 1651 cm^−1^ are from citric acid anhydrous. The peak at 1077 cm^−1^ is assigned to the C–O stretching vibration. All these characteristic peaks demonstrate that the obtained CDs are highly hydrophilic due to the rich carboxyl and hydroxyl groups on the surface. The FTIR spectra of MIP@CDs and NIP@CDs are of no significant difference considering the similar preparation procedures and starting materials. The vibration signals of Si–O are found at 792 cm^−1^ and 452 cm^−1^. The characteristic peaks at 2937 cm^−1^ and 1482 cm^−1^ can be assigned to the stretching vibration of CH_2_–N and C–H, respectively, conforming the existence of amide group. The peak at 1560 cm^−1^ is from the stretching vibration of N–H from aminopropyl group.^47^ These experimental results reveal that the silica layer was formed on the surface of both MIP@CDs and NIP@CDs. XPS spectra of MIP@CDs and NIP@CDs both exhibit five peaks at 102.1, 153.1, 284.4, 398.9 and 531.7 eV, which are attributed to Si 2p, Si 2s, C 1s, N 1s, and O 1 s, respectively, confirming the existence of the silica layer(Fig. S2†).

The optical properties of CDs were examined by UV-vis absorption and fluorescence spectra (Fig. S3†). The as-prepared CDs exhibit an absorption peak around 360 nm and a maximum emission peak around 448 nm (Fig. S3a†). Furthermore, the maximum emission peak showed red-shift with increased excitation wavelength, demonstrating the unique characteristic of CDs. After being functionalized with silica layer, both MIP@CDs and NIP@CDs show the same absorption peak at 360 nm and emission peak at 448 nm as CDs (Fig. S3b and c†), thus the imprinted polymer layer doesn't alter the optical properties of CDs. The stability of MIP@CDs was also tested. As shown in Fig. S3d,† the emission intensity of MIP@CDs at 448 nm remained roughly unchanged within 14 days, illustrating an excellent stability.

**Fig. 2:**
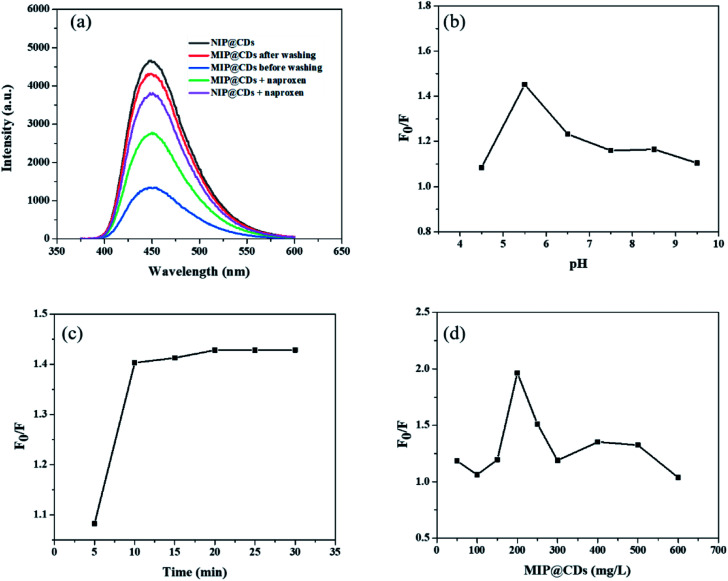
(a) The emission spectra of MIP@CDs and NIP@CDs. (pH = 5.5, *λ*_ex_ = 360 nm, the concentrations of MIP@CDs and NIP@CDs were 200 mg L^−1^). Fluorescence quenching ratios of MIP@CDs with different (b) pH values; (c) incubation time; (d) concentrations of MIP@CDs. *λ*_ex_ = 360 nm.

Preliminary evaluation on the selective recognition of naproxen by MIP@CDs was also carried out. As shown in Fig. 2a, compared to NIP@CDs, the fluorescence intensity of MIP@CDs before removing the template naproxen molecules was quite weak. When naproxen was washed away by methanol, the fluorescence was almost completely recovered. The emission intensity dropped again when naproxen (14 μL, 50 μM) was added. However, the addition of naproxen doesn't change the fluorescence intensity of NIP@CDs. These results demonstrate that MIP@CDs can detect naproxen through the change of fluorescence intensity, and NIP@CDs without imprinted polymer show no response to naproxen.

### 3.2 Optimization of detection conditions

In order to achieve high sensitivity for naproxen recognition, detection conditions, such as pH value, incubation time and concentration of MIP@CDs solution, were optimized. The effect of pH on the fluorescence quenching behaviour of naproxen for MIP@CDs was investigated by using PBS buffer to adjust pH. As shown in Fig. 2b, the fluorescence quenching ratio at 448 nm of MIP@CDs by naproxen first increased with increasing pH values from 4.4 to 5.5, then decreased from 5.5 to 9.5. The reduced sensitivity at lower or higher pH is ascribed to the proton protonation in acid environment and ionization in alkaline environment, both of which will cause structural damage of the recognition cavities in the silica shell, thus leading to a decreased response sensitivity to naproxen.^48^ The effect of incubation time was also explored, Fig. 2c shows that the fluorescence quenching efficiency by naproxen to MIP@CDs reached a maximum after 10 min and remained relatively stable with further prolonging time to 30 min, which means a relatively completed binding between naproxen and the imprinted cavities of MIP@CDs occurred within 10 min. The amount of MIP@CDs used for the quenching experiment was also optimized (Fig. 2d). The highest fluorescence quenching ratio was observed at the concentration of 200 mg L^−1^ within the range from 50 mg L^−1^ to 600 mg L^−1^. On the basis of the above experiments for optimization of detection conditions, pH of 5.5, the incubation time of 10 min and 200 mg L^−1^ of MIP@CDs were selected for the subsequent studies.

### 3.3 Analytical performance for the detection of naproxen

**Fig. 3:**
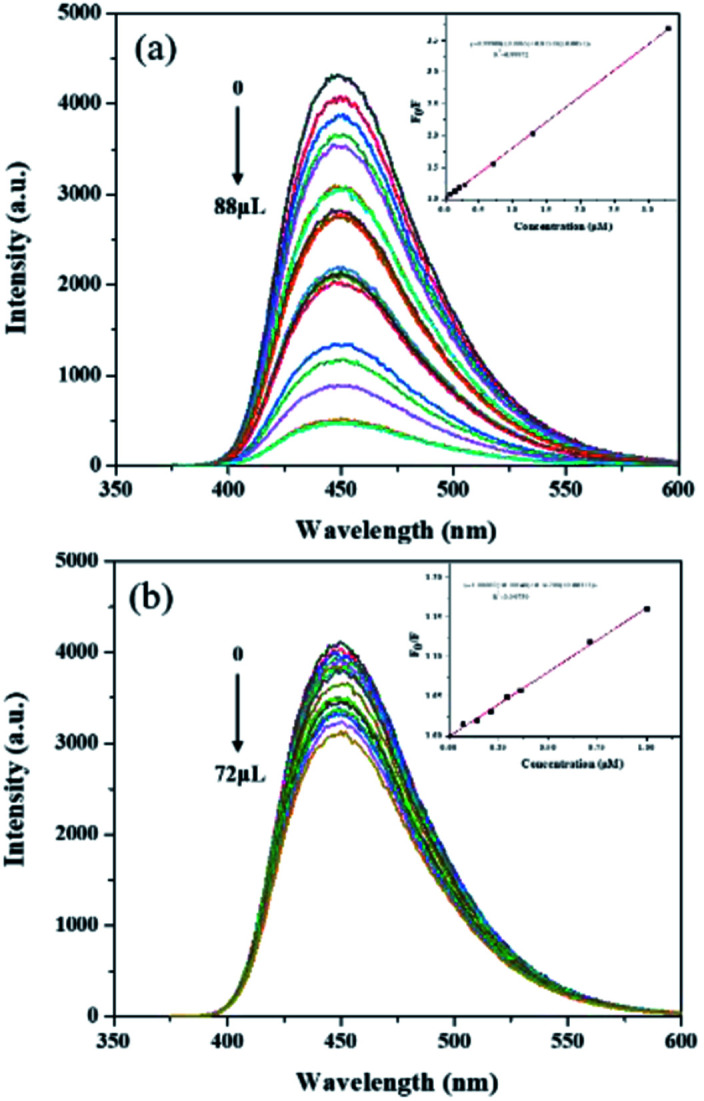
Fluorescence spectra and Stern–Volmer plots of (a) MIP@CDs and (b) NIP@CDs with different volumes of naproxen (*λ*_ex_ = 360 nm). Inset: Stern–Volmer equations of MIP@CDs and NIP@CDs.

The analytical performance of MIP@CDs for the detection of naproxen was investigated under the optimized conditions. As shown in Fig. 3a, the fluorescence spectra of MIP@CDs were collected with different amounts of naproxen solution (50 μM) to investigate its quenching behaviour. The fluorescence intensity of MIP@CDs decreased distinctly with increasing the amount of naproxen, whereas no obvious fluorescence intensity change was observed for NIP@CDs upon adding naproxen. The better response sensitivity of MIP@CDs attributes to the existence of imprinted cavities in the silica shell, which provides selective recognition and binding affinity to naproxen molecules.^49^ The quenching efficiency of naproxen molecules was then quantified by the following Stern–Volmer equation:

1 *F*_0_/*F* = 1 + *K*_SV_*C*

where *F*_0_ and *F* are the fluorescent intensities at 448 nm of MIP@CDs in the absence and presence of naproxen, respectively, *C* is the concentration of naproxen, and *K*_SV_ is the Stern–Volmer quenching constant. A good linear relationship between *F*_0_/*F* (quenching ratio) and different concentration of naproxen was observed, and the linear regression equation was obtained as *F*_0_/*F* = 1 + 0.815*C*_[naproxen]_, where *K*_SV-MIP_ was determined as 8.15 × 10^5^ M^−1^. As a comparison, the Stern–Volmer equation for NIP@CDs was also determined as *F*_0_/*F* = 1 + 0.162*C*[_naproxen_] with *K*_SV-NIP_ of 1.62 × 10^5^ M^−1^ (Fig. 3b). The imprinting factor (IF), defined as IF = *K*_SV-MIP_/*K*_SV-NIP_, is an important parameter to evaluate the selectivity of imprinted polymer. Here IF was calculated to be 5, which suggests an excellent selectivity towards to naproxen by MIP@CDs. Limit of detection for MIP@CDs was also calculated to be 0.03 μM based on the ratio of signal-to-noise of 3, which is close to or lower than that of the existing sensors for naproxen, as shown in Table S2.†^50,51^ These detailed studies demonstrate that the developed MIP@CDs sensor shows a wide linear range response (0.05 ∼ 4 μM) with a low limit of detection (0.03 μM) to naproxen.

Given the strong hydrogen bonding reaction between the carboxyl group of naproxen and amino group of the imprinted cavities, the fluorescence quenching mechanism may possibly be speculated as energy transfer or charge transfer between naproxen and MIP@CDs. Since there was no obvious overlap between the absorption spectra of naproxen and the emission spectrum of MIP@QDs (Fig. S4a†), energy transfer cannot happen to induce fluorescence quenching. As previously reported,^52^ charges in the conductive bands of QDs could transfer to a lowest unoccupied orbital of analyte molecules, thus charge transfer is considered as the main reason for fluorescence quenching. Lifetimes of MIP@CDs in the absence and presence of naproxen were also measured (Fig. S4b†). The lifetime of MIP@CDs decreased from 17.17 ns to 14.34 ns after adding naproxen, which means dynamic quenching occurred. The collisional encounters and weak bonding reaction between MIP@CDs and naproxen depopulates the excited state of MIP@CDs, causing the decreasing of lifetime.

### 3.4 Selectivity and anti-interference

**Fig. 4:**
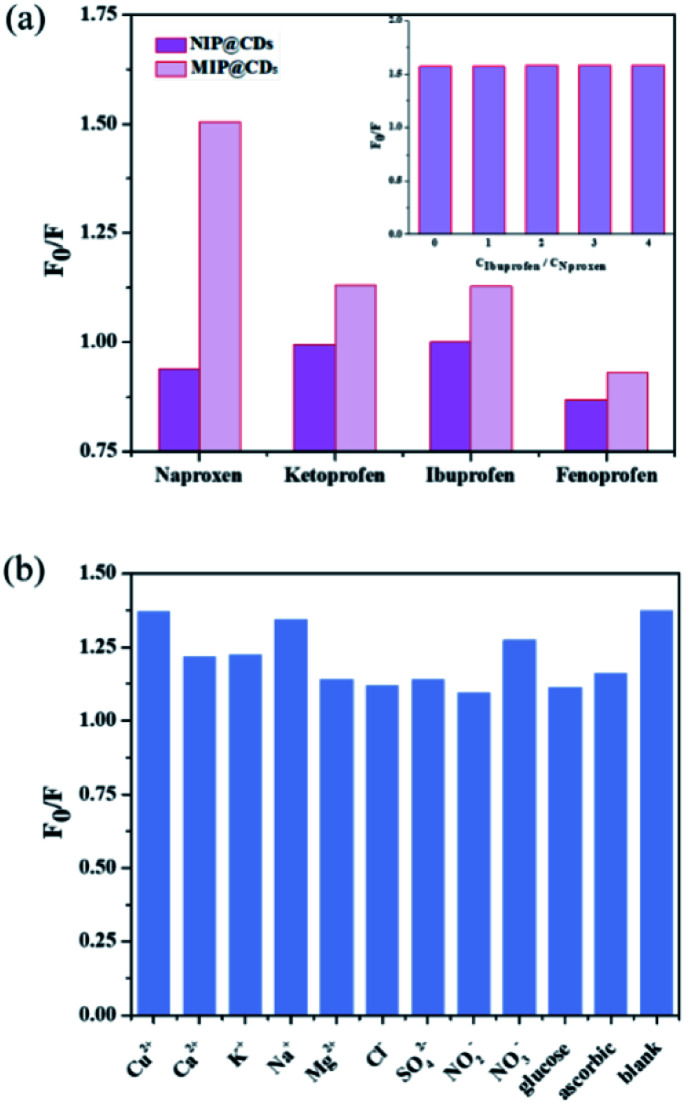
(a) Fluorescence response of MIP@CDs and NIP@CQDs for naproxen and its structural analogs (the concentration of naproxen, ketoprofen, ibuprofen, and fenoprofen was 1 μM). Inset: competitive binding to MIP@CDs from ibuprofen under different concentrations. (b) Fluorescence response of MIP@CDs in the presence of interfering substances (the concentrations of naproxen and interfering substances were 1 and 500 μM, respectively). *λ*_ex_ = 360 nm, *λ*_em_ = 448 nm.

The selectivity and anti-interference performances of MIP@CDs were also investigated. Functional and structural analogs (ibuprofen ketoprofen, and fenoprofen) were selected for the selectivity experiments. As shown in Fig. 4a, MIP@CDs exhibited a much higher fluorescence quenching efficiency towards naproxen than that of its analogs, due to the existence of imprinted cavities with the exact shape and size complementary to template molecules. Meanwhile, NIP@CDs without imprinted cavities displayed similar inefficient quenching behaviours for naproxen and analogs, which highlights the necessity of imprinted cavities for the selectivity performance. Ibuprofen was chosen for competitive binding experiment to evaluate the anti-interference ability of MIP@CDs. With increasing the *C*_IBU_/*C*_NAPROXEN_ ratio from 0 to 4, the fluorescence quenching ratio of MIP@CDs remained unchanged by naproxen, illustrating no competitive binding from ibuprofen and high affinity of naproxen to MIP@CDs.

Considering the coexistence of interfering substance in real water samples, the fluorescence quenching performances of naproxen to MIP@CDs in the presence of common metal ions, inorganic salt ions, and potentially existing organics (such as Cu^2+^, Ca^2+^, K^+^, Na^+^, Mg^2+^, Cl^−^, SO_4_^2−^, NO_3_^−^, NO_2_^−^), were investigated. As illustrated in Fig. 4b, the quenching ratios of naproxen displayed no obvious change while coexisting with interfering substances. These results further demonstrated that MIP@CDs exhibited excellent anti-interference property and could be applied to selectively detect naproxen molecule in practical applications.

### 3.5 Practical applications

To assess the practical application of MIP@CDs in real samples, recovery studies were performed by spiking the real samples (tap water, river water, and simulative human urine) with various amounts of naproxen. The averaged recoveries with relative standard deviation (RSD, *n* = 3) for each concentration are listed in Table 1. Satisfactory recoveries with low RSDs were obtained for all three real samples, demonstrating the prepared MIP@CQD sensor can be utilized for accurate determination of trace naproxen in complicated real samples.

**Table 1:** Results for the detection of naproxen in real samples

Samples	Spiked (μM)	Found (μM)	Recovery (%)	RSD (%)
Tap water	0.5	0.48	96.0	4.2
	1	1.04	104.0	3.6
	1.5	1.37	91.3	9.7
	0.5	0.51	102.0	4.7
River water	1	1.13	113.0	7.4
	1.5	1.44	96.0	4.7
	0.5	0.46	92.0	7.7
Simulative urine	1	0.95	95.0	8.4
	1.5	1.52	101.3	5.4

**Fig. 5:**
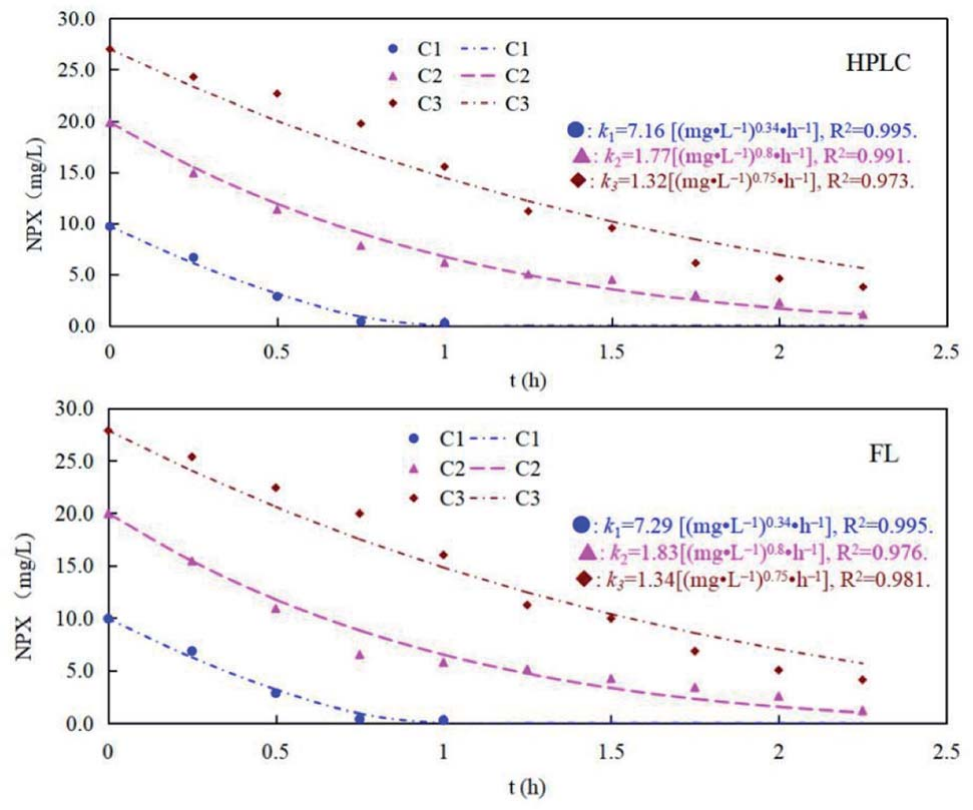
The degradation rates of different concentrations of naproxen under UV light monitored by HPLC and MIP@CDs (C1 = 10 mg L^−1^, C2 = 20 mg L^−1^, and C3 = 10 mg L^−1^).

Naproxen may also generate intermediates when exposed to sunlight irradiation (UV degradation) in natural water, which complicates the analysis of its existence. Thus, we also investigated the detection performance of MIP@CDs for naproxen under UV light. As shown in Fig. 5, the concentration of naproxen solution decreased under UV irradiation for all three samples, and the degradation curve is accorded with the first-order degradation curve. There is no significant difference between HPLC and MIP@CDs test results for detection of naproxen concentration, indicating that the intermediates produced during the UV degradation have no effects on the fluorescence response of MIP@CDs to naproxen.

## 4. Conclusions

In summary, we have successfully developed a fluorescent CDs sensor for specific recognition and sensitive detection of naproxen molecules based on molecularly imprinted polymer. The fluorescence of the obtained MIP@CDs was selectively quenched by naproxen, and a low LOD of 0.03 μM with a linear range of 0.05–4 μM was achieved. Anti-interference performance in the presence of coexisting substances and recovery studies of the developed MIP@CDs sensor in real samples were also investigated to reveal a potential application for practical naproxen monitoring and evaluation in the environment.

## Author contributions

Ke Li: investigation, validation, analysis, writing of the original draft. Min Zhang: investigation. Xingyu Ye: investigation. Yongming Zhang: review and editing. Guisheng Li: conceptualization. Rui Fu: review and editing. Xiaofeng Chen: conceptualization, supervision, funding acquisition, writing, review and editing.

## Conflicts of interest

There are no conflicts to declare.

## Supplementary Material

RA-011-D1RA04817A-s001
