# Supplementary material for: Highly sensitive and selective detection of naproxen via molecularly imprinted carbon dots as a fluorescent sensor
Source: RSC Adv. 2021 Aug 31;11(46):29073–9. doi: 10.1039/d1ra04817a (PMC9038106; doi:10.1039/d1ra04817a)
Supplement: RA-011-D1RA04817A-s001 [file RA-011-D1RA04817A-s001.pdf]

Electronic Supplementary Material (ESI) for RSC Advances.
This journal is © The Royal Society of Chemistry 2021

Supporting information

Highly Sensitive and Selective Detection of Naproxen via Molecularly Imprinted Carbon Dots as a Fluorescent Sensor

Ke Li[a], Min Zhang[a], Xingyu Ye[b], Yongming Zhang[a], Guisheng Li[b], Rui Fu[c], and Xiaofeng Chen*[a]

[a] *School of Environmental and Geographical Sciences, Shanghai Normal University, Shanghai 200234, China. E-mail: xiaofengchen@shnu.edu.cn*
[b]*Education Ministry Key and International Joint Lab of Resource Chemistry, Shanghai Key Laboratory of Rare Earth Functional Materials, Shanghai Normal University, Shanghai 200234, China.*
[c]*RNA Bioscience Initiative, University of Colorado School of Medicine, Aurora, CO, 80045, USA.*

Table S1. The preparation recipe of human urine sample.

Compound	Mass or Volume
NaCl	0.52 g
$CaCl_2$	0.028 g
$MgSO_4$	0.24 g
KH_2PO_4	0.142 g
KH_2PO_4	0.16 g
$C_6H_8O_7$	1.5 g
$CH_3CHOHCOOH$	0.009 g
$CO(NH_2)_2$	1.021 g
NH_4Cl	0.134 g
$NaHCO_3$	0.21 g
H_2O	100 mL

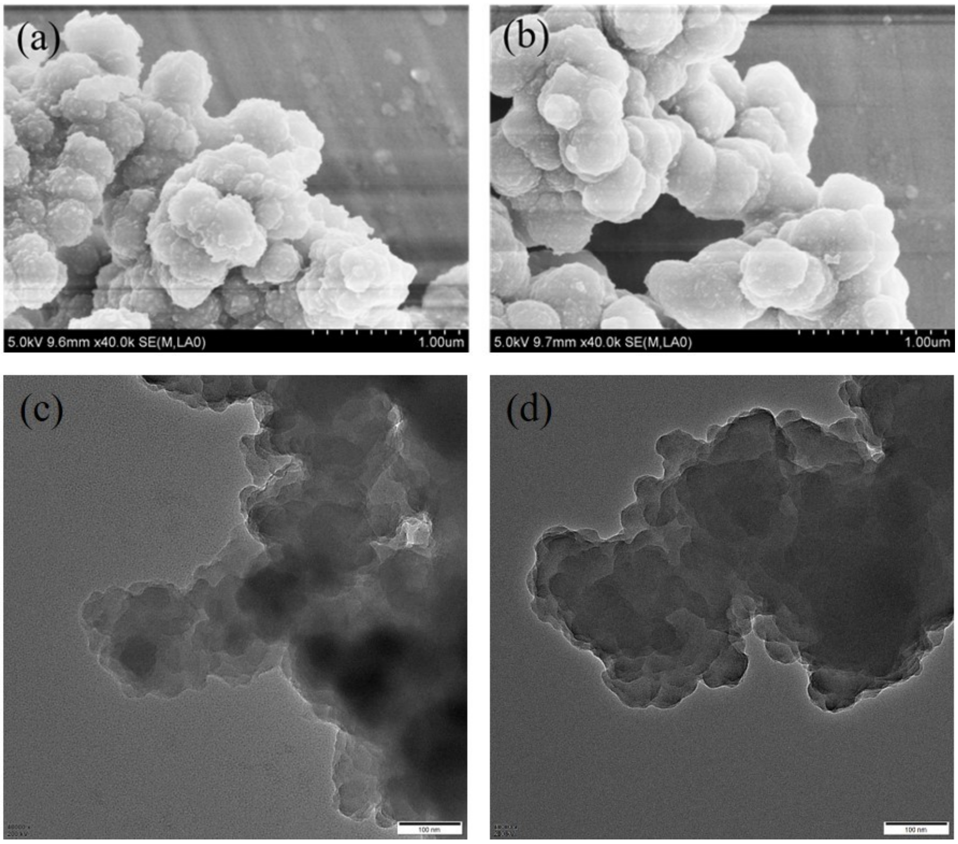

Fig. S1. SEM and TEM images of (a, c) MIP@CDs; (b, d)NIP@CDs.

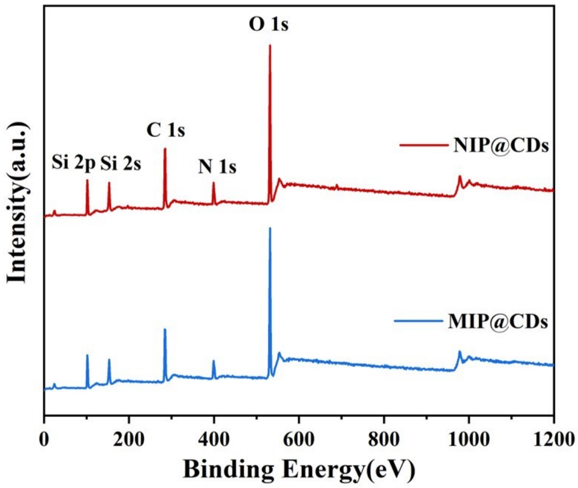

Fig. S2. The XPS survey scans of MIP@CDs and NIP@CDs.

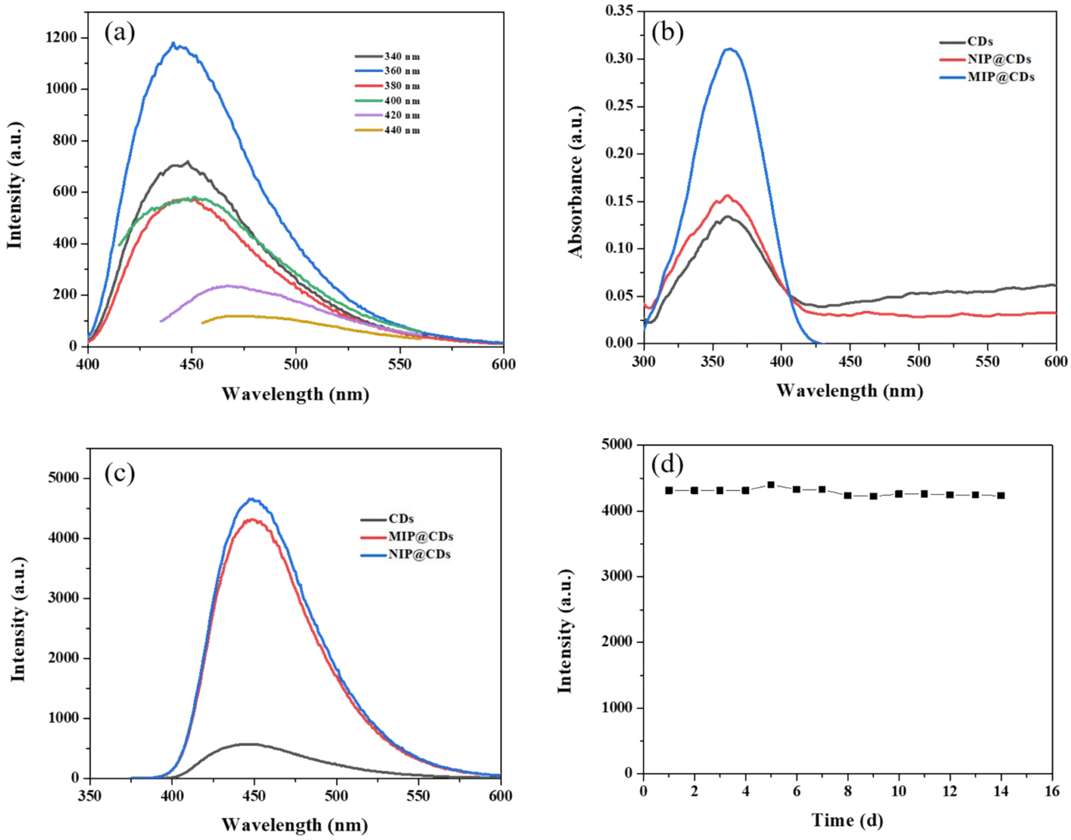

Fig. S3. (a) The emission spectra of the CDs in ethanol with different excitation wavelengths; Absorption (b) and emission (c) spectra of CDs, MIP@CDs, NIP@CDs; (d) The emission intensity of MIP@CDs (280 mg/L) at 448nm within 14 days.

Table S2 Different methods for naproxen detection.

Method	Materials	LOD	Linear range	references
Electrochemical method	AgNPs@GO-β-CD	0.08 μM	0.4-80 μM	1
Chiral electrochemical method	L- CYS/AuNPs/Au E	0.67 μM	2-20 μM	2
Photoelectrochemical method	$BiVO_4$/CuO	5 nM	5-480 nM	3
Plasmon method	Tβ-CD-Au NPs	0.6 ug/L	4-180 ug/L	4
Electrochemical method	$FeNi_3$/CuS/BiOCl nanocomposite	0.06 μM	0.2-500 μM	5
Microextraction method	Hollow fiber liquid	1.3 ng/L	4-300 ng/L	6
Fluorescent method	MIP@CDs	0.03 μM	0.05-4 μM	this work

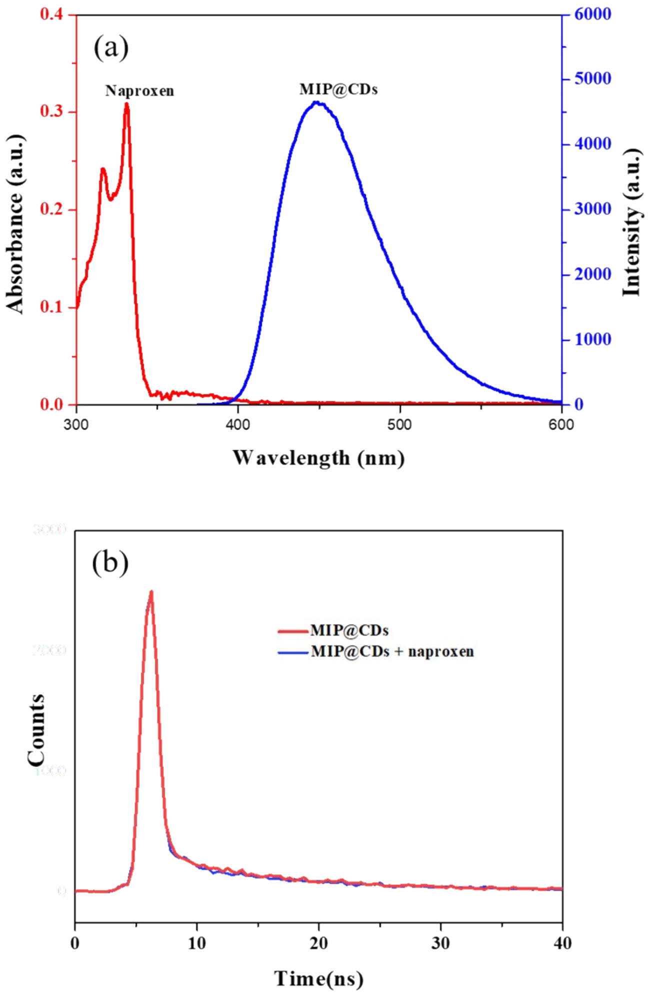

Fig. S4. (a) Absorption spectra of naproxen and emission spectra of MIP@CDs; (b) Fluorescence decay traces of MIP@CDs in the absence and presence of naproxen.

References:

1 S. Tarahomi, G. H. Rounaghi, L. Daneshvar, *Sens. Actuators B Chem.*, 2019, **286,** 445-450.
2 A. Afkhami, F. Kafrashi, M. Ahmadi, T. Madrakian, *RSC Adv.*, 2015, **5,** 58609-58615.

3 T. M. Prado, C. C. Badaró, R. G. Machado, S. FadiniP, O, Fatibello-Filho, F. C. Moraes, *Electroanalysis*, 2020, **32**, 1930-1937.
4 J. Khodaveisi, A. M. H. Shabani, S. Dadfarnia, D. A. Saberi, *Spectrochim Acta A Mol Biomol Spectrosc*., 2017, **179**, 11-16.
5 P. M. Jahani, H. A. Javar, H. Mahmoudi-Moghaddam, *J Mater Sci: Mater Electron*, 2020, **31**, 14022-14034.
6 M. Asadi, S. Dadfarnia, A. M. H. Shabani, B. Abbasi, *Food Anal. Methods*, 2016, **9**, 2762-2772.
